# Dispositivos Cardíacos Eletrônicos Implantáveis em Chagásicos e Não-Chagásicos: Um Olhar Crítico sobre o Prognóstico

**DOI:** 10.36660/abc.20240629

**Published:** 2024-11-08

**Authors:** Fernanda Almeida Andrade

**Affiliations:** 1 Hospital das Clínicas da Faculdade de Medicina da Universidade de São Paulo Instituto do Coração São Paulo SP Brasil Instituto do Coração do Hospital das Clínicas da Faculdade de Medicina da Universidade de São Paulo, São Paulo, SP – Brasil; 2 Universidade Federal de São Paulo Escola Paulista de Medicina São Paulo SP Brasil Escola Paulista de Medicina da Universidade Federal de São Paulo (EPM/UNIFESP), São Paulo, SP – Brasil

**Keywords:** Doença de Chagas, Marca-Passo Artificial, Terapia de Ressincronização Cardíaca, Cardiomiopatia Dilatada

A doença de Chagas tem como uma de suas principais complicações o acometimento do sistema cardiovascular, causando lesão crônica ao miocárdio e ao sistema de condução intracardíaco, além de arritmias e morte súbita; ela foi descrita pela primeira vez em 1909 e continua sendo um desafio de saúde na América do Sul acometendo cerca de 8 milhões de pessoas, contudo não está mais confinada ao nosso território, com casos emergindo nos EUA, Europa, Canadá, Austrália e Japão.^1,2^ Quando se compara a cardiomiopatia chagásica (CCh) com outras etiologias de cardiomiopatia dilatada, os dados revelam um prognóstico mais reservado e maior morbidade e mortalidade dos pacientes afetados.^2,3^

No Brasil, onde a doença de Chagas ainda representa um problema de saúde pública relevante, muitos pacientes com CCh são submetidos ao implante de dispositivos eletrônicos cardíacos, como marca-passos (MP) e ressincronizadores cardíacos (TRC).^4^ A presença de cicatrizes miocárdicas e o envolvimento do sistema de condução são características bem descritas dessa cardiopatia, justificando o implante de dispositivos como o MP para controle de bradiarritmias ou o TRC para insuficiência cardíaca avançada. Apesar do avanço no tratamento dessas condições, existem lacunas sobre o impacto a longo prazo desses dispositivos em pacientes chagásicos *versus* não chagásicos.^5,6^

Historicamente, os pacientes com CCh apresentam pior prognóstico em comparação a outras cardiomiopatias dilatadas. Estudos anteriores associaram a CCh a uma maior mortalidade devido à progressão da insuficiência cardíaca, arritmias graves e morte súbita cardíaca (responsável por 55 a 65% das mortes nesses pacientes).^5,7–9^ No entanto, recentemente, um estudo de coorte^10–12^ apresentou dados que desafiam o paradigma vigente, mostrando que a presença de doença de Chagas (23,4%) não foi um fator preditivo de maior mortalidade (HR: 1,14, IC: 95%, 0,86-1,51, p=0,365) ou hospitalizações (HR: 0,79, IC: 95%, 0,61-1,04, p=0,09).^1^

## Fatores que podem explicar os resultados

Uma possível explicação para a ausência de diferença significativa no prognóstico entre pacientes chagásicos e não chagásicos pode estar relacionada à melhoria na gestão clínica e na estruturação dos cuidados de saúde para essa população ao longo dos últimos anos.^6,11^ O estudo foi conduzido em um centro terciário de referência, com provável acesso à tecnologia de ponta e acompanhamento especializado.^1^ Programas de seguimento ambulatorial especializado e adesão aos tratamentos recomendados podem ter reduzido as disparidades de desfechos entre os dois grupos.^11^

Além disso, o estudo analisou dados de pacientes ao longo de três eras diferentes, o que permitiu a avaliação de possíveis mudanças ao longo do tempo.^1^ O fato de não haver diferenças de mortalidade entre chagásicos e não chagásicos em cada uma dessas eras reforça a ideia de que o progresso no tratamento, tanto clínico quanto cirúrgico, pode ter diluído o impacto prognóstico adverso previamente atribuído à CCh.^1,12^

Outro ponto a ser considerado é o perfil dos pacientes incluídos, embora a idade média dos pacientes chagásicos tenha sido inferior à dos não chagásicos no momento do implante (65 *vs.* 69 anos, respectivamente), a mortalidade ajustada por idade não mostrou diferença significativa.^1^ Esse achado sugere que o manejo terapêutico adequado, independentemente da etiologia subjacente da cardiopatia, pode ser um fator de maior influência sobre os desfechos do que a própria etiologia em si.^1^

## Limitações e perspectivas futuras

Esse contraste entre os trabalhos pode ser explicado por diferentes desenhos de estudo, populações investigadas e melhorias nos cuidados ao longo do tempo, principalmente com relação à terapia medicamentosa de insuficiência cardíaca.^1,8^ Estudos realizados em contextos de menor acesso a cuidados de saúde especializados ou em fases anteriores da evolução tecnológica dos dispositivos cardíacos podem ter mostrado desfechos mais adversos.^7,9^ No estudo de Vasconcelos et al.^1^ é notável que uma pequena proporção de pacientes chagásicos recebeu dispositivos de ressincronização (3,3%), enquanto essa proporção foi muito maior entre os não chagásicos (18,1%), o que pode refletir uma menor indicação de TRC para chagásicos devido a fatores como menor resposta à terapia de ressincronização.^1,4^

A alta incidência de arritmias ventriculares e morte súbita são características distintivas da CCh, como relatado por Vasconcelos et al., que observaram pior evolução clínica em pacientes chagásicos com MP.^1^ Da mesma forma, a insuficiência cardíaca avançada, que responde por uma grande parcela dos óbitos em pacientes com CCh, é outro fator que frequentemente piora o prognóstico em comparação a outras cardiomiopatias dilatadas.^3,5^ Além disso, Martinelli Filho et al. mostrou que pacientes com CCh submetidos a TRC têm pior prognóstico em comparação aos pacientes tratados com TRC com cardiomiopatia dilatada por outras causas; este é um estudo importante com a maior série publicada descrevendo pacientes com CCh tratados com TRC.^12^

A crítica deve existir a respeito da existência de limitações que podem influenciar nos resultados, como a qualidade dos dados registrados nos prontuários eletrônicos (o que pode não capturar completamente as nuances clínicas de cada caso), terapia medicamentosa utilizada, a quantidade de fibrose correlacionada à região onde o eletrodo do ventrículo esquerdo está posicionado, e adesão ao tratamento pelos pacientes.^1,12^ Outra limitação relevante foi a exclusão dos pacientes com cardiodesfibrilador implantável isolado, dispositivo frequentemente implantado em pacientes com maior risco de arritmias graves e morte súbita. Embora essa exclusão tenha sido feita para reduzir vieses, ela também limita a generalização dos resultados para a população total de pacientes com dispositivos eletrônicos cardíacos implantáveis.^1,6,10–12^

De toda forma, esses achados são encorajadores e reforçam a importância de uma gestão clínica eficiente, que pode mitigar as diferenças prognósticas associadas à etiologia das cardiomiopatias.^7,11^ Contudo, é crucial continuar investigando essa população em diferentes contextos e com métodos prospectivos com maior detalhamento das características clínicas, laboratoriais e terapêuticas das populações estudadas para validar esses achados e, eventualmente, redefinir o manejo desses pacientes.^13^
