# Supplementary material for: Dispositivos Cardíacos Eletrônicos Implantáveis em Chagásicos e Não-Chagásicos: Um Olhar Crítico sobre o Prognóstico
Source: Arq Bras Cardiol. 2024 Nov 8;121(9):e20240629. [Article in Portuguese] doi: 10.36660/abc.20240629 (PMC11634229; doi:10.36660/abc.20240629)
Supplement: Translation

# Translation: Cardiac Implantable Electronic Devices in Chagasic and Non-Chagasic Patients: A Critical Look at the Prognosis

**DOI:** 10.36660/abc.20240629i

**Authors:** Fernanda Almeida Andrade

**Affiliations:** 1 São Paulo SP Brazil Instituto do Coração do Hospital das Clínicas da Faculdade de Medicina da Universidade de São Paulo, São Paulo, SP – Brazil; 2 São Paulo SP Brazil Escola Paulista de Medicina da Universidade Federal de São Paulo (EPM/UNIFESP), São Paulo, SP – Brazil

**Keywords:** Chagas Disease, Artificial Pacemaker, Cardiac Resynchronization Therapy, Dilated Cardiomyopathy

Chagas disease has as one of the main complications of Chagas disease is the involvement of the cardiovascular system, causing chronic damage to the myocardium and intracardiac conduction system, as well as arrhythmias and sudden death. It was first described in 1909 and continues to be a health challenge in South America, affecting around 8 million people. However, it is no longer confined to our territory, with cases emerging in the USA, Europe, Canada, Australia, and Japan.^1,2^ When comparing Chagas cardiomyopathy (ChC) with other etiologies of dilated cardiomyopathy, data reveal a more reserved prognosis and greater morbidity and mortality in affected patients.^2,3^

In Brazil, where Chagas disease still represents a relevant public health problem, many patients with ChC undergo the implantation of electronic cardiac devices, such as pacemakers (PM) and cardiac resynchronization devices (CRD).^4^ The presence of myocardial scarring and involvement of the conduction system are well-described characteristics of this cardiopathy, justifying the implantation of devices such as PM to control bradyarrhythmias or CRD for advanced heart failure. Despite advances in the treatment of these conditions, there are gaps regarding the long-term impact of these devices in Chagasic *versus* non-Chagasic patients.^5,6^

Historically, patients with ChC have a worse prognosis compared with other dilated cardiomyopathies. Previous studies have associated ChC with increased mortality due to the progression of heart failure, serious arrhythmias, and sudden cardiac death (accounting for 55 to 65% of deaths in these patients).^5,7–9^ However, a recent cohort study^10–12^ presented data that challenged the current paradigm, showing that the presence of Chagas disease (23.4%) was not a predictor of increased mortality (HR: 1.14, 95% CI, 0.86-1.51, p=0.365) or hospitalizations (HR: 0.79, 95% CI, 0.61-1.04, p=0.09).^1^

## Factors that may explain the results

One possible explanation for the lack of significant difference in prognosis between patients with and without Chagas disease may be related to improvements in clinical management and the structuring of health care for this population over the past few years.^6,11^ The study was conducted in a tertiary referral center, with likely access to cutting-edge technology and specialized monitoring.^1^ Specialized outpatient follow-up programs and adherence to recommended treatments may have reduced the disparities in outcomes between the two groups.^11^

Furthermore, the study analyzed patient data across three different eras, which allowed for the assessment of possible changes over time.^1^ The fact that there were no differences in mortality between chagasic and non-chagasic patients in each of these eras reinforces the idea that progress in treatment, both clinical and surgical, may have diluted the adverse prognostic impact previously attributed to ChC.^1,12^

Another point to be considered is the profile of the patients iincluded, although the mean age of chagasic patients was lower than that of non-chagasic patients at the time of implantation (65 *vs.* 69 years, respectively), age-adjusted mortality showed no significant difference.^1^ This finding suggests that appropriate therapeutic management, regardless of the underlying etiology of the heart disease, maybe a factor with greater influence on outcomes than the etiology itself.^1^

## Limitations and future perspectives

Different study designs can explain this contrast between studies, populations investigated, and improvements in care over time (especially with regard to drug therapy for heart failure).^1,8^ Studies conducted in contexts with less access to specialized health care or in earlier phases of the technological evolution of cardiac devices may have shown more adverse outcomes.^7,9^ In the study by Vasconcelos et al.,^1^ it is notable that a small proportion of chagasic patients received resynchronization devices (3.3%), while this proportion was much higher among non-chagasic patients (18.1%), this may reflect a lower indication of CRD for chagasic patients due to factors such as lower response to resynchronization therapy.^1,4^

The high incidence of ventricular arrhythmias and sudden death are distinctive features of ChC, as reported by Vasconcelos et al., who observed worse clinical outcomes in Chagas disease patients with PM.^1^ Similarly, advanced heart failure, which accounts for a large proportion of deaths in patients with ChC, is another factor that often worsens the prognosis compared with other dilated cardiomyopathies.^3,5^ Moreover, Martinelli Filho et al. showed that patients with CCh who underwent cardiac resynchronization therapy (CRT) have a worse prognosis compared to patients treated with CRT for dilated cardiomyopathy from other causes; this is an important study with the largest published series describing patients with CCh treated with CRT.^12^

Criticism should be given to the existence of limitations that may influence the results, such as the quality of the data recorded in the electronic medical records (which may not fully capture the clinical nuances of each case), drug therapy used, and patient adherence to treatment, the amount of fibrosis related to the area where the left ventricular electrode is positioned, and patient adherence to treatment.^1,12^ Another relevant limitation was the exclusion of patients with an isolated implantable cardioverter-defibrillator, a device frequently implanted in patients at higher risk of serious arrhythmias and sudden death. Although this exclusion was made to reduce bias, it also limits the generalization of the results to the total population of patients with cardiac implantable electronic devices.^1,6,10–12^

In any case, these findings are encouraging and reinforce the importance of efficient clinical management, which can mitigate the prognostic differences associated with the etiology of cardiomyopathies.^7,11^ However, it is crucial to continue investigating this population in different contexts and with prospective methods with greater detail of the clinical, laboratory, and therapeutic characteristics of the studied populations to validate these findings and, eventually, redefine the management of these patients.^13^
